# From frailty to resilience: exploring adaptive capacity and reserve in older adults–a narrative review

**DOI:** 10.3389/fragi.2025.1520842

**Published:** 2025-07-01

**Authors:** Caglar Cosarderelioglu, Jeremy D. Walston, Peter M. Abadir

**Affiliations:** Johns Hopkins University School of Medicine, Division of Geriatric Medicine and Gerontology, Baltimore, MD, United States

**Keywords:** Reserve, frailty, disability, older adults, Alzheimer’s disease, psychosocial resilience, physical resilience, cognitive resilience

## Abstract

Resilience, the capacity to adapt and recover from adversity, plays a critical role in the health and wellbeing of older adults. In geriatric populations, resilience encompasses physical, cognitive, and psychosocial domains and is essential for maintaining functional independence and quality of life amidst the challenges of aging. This review explores the concept of resilience within geriatric medicine across physical, cognitive, and psychosocial domains, highlights the differences from frailty and reserve, underscores importance of stressors, summarizes key biomarkers that predict resilience, and evaluates interventions designed to enhance resilience in older adults.

## 1 Introduction

As human lifespan increases, managing frailty and enhancing resilience becomes increasingly critical. With longer life comes the heightened risk of age-related conditions, which typically accumulate and manifest more prominently in later life. There is a period in life when the risk of frailty and disability begins to increase rapidly. Olshansky referred to this phase as the “red zone,” a period during which traditional disease-focused interventions become increasingly challenging and diseases tend to become more resistant, making it much harder to manage them effectively with conventional approaches (Olshansky, 2018). In the early 20th century, most deaths occurred before this stage, but by 2016, a significant portion of deaths shifted toward advanced ages (Olshansky, 2018; HMD, 2024; OACT, 2024). Aging science now aims to delay the onset of this “red zone” and compress it, extending the years of healthy, independent living. By doing so, we can mitigate the growing burden of frailty, decrease functional decline, and maintain resilience in an aging population. The geroscience hypothesis suggests that the core biological processes of aging are the main drivers of chronic illnesses, multiple health conditions, and ultimately death (Sierra and Kohanski, 2017). In line with this, it has been suggested that strong resilience to health stressors during early-to-mid-life may indicate healthy aging, whereas reduced resilience could signal accelerated aging, even before noticeable signs of organ or physiological dysfunction emerge (LeBrasseur, 2017). This emphasizes the need to develop interventions that enhance biological and psychological resilience, reducing the impact of age-related stressors and chronic diseases. Addressing these challenges will be key to ensuring that longer lifespans are accompanied by better health and quality of life—what is referred to as increasing healthspan—a critical focus for aging research and public health efforts.

In this context, it becomes crucial to deepen our understanding of the reserve capacity of older adult (Colón-Emeric et al., 2019). Such understanding is vital for identifying the older adults who are most vulnerable to stressors and for creating targeted treatments and preventive strategies to improve their overall health (Whitson et al., 2018). One established approach for assessing static reserve at a given time point has been the concept of frailty (Fried et al., 2001). Frailty encompasses a state of heightened vulnerability to stressors due to declines in physiological function across multiple systems, making it a valuable indicator of an individual’s reserve capacity.

However, frailty alone does not capture the full picture of how individuals adapt to stress over time. With advancements in defining different aspects of frailty and its clinical implications, the concept of resilience has emerged as a pivotal framework for understanding how older adults navigate stressors, including cognitive, psychosocial, and physical challenges such as bereavement, chronic illnesses, and financial difficulties. Resilience is increasingly recognized as a critical determinant of health outcomes among older adults, reflecting their capacity to recover and rebound from these various stressors (Walston et al., 2023). Unlike frailty, which focuses on a more static assessment of vulnerability, resilience highlights the dynamic and adaptive responses to challenges.

This review paper explores the current understanding of resilience in gerontology and geriatric medicine, distinguishing it from frailty—a related but distinct concept—and synthesizing insights from significant studies in the field. By enhancing our understanding of both frailty and resilience, we can develop more comprehensive approaches to aging that not only address the biological vulnerabilities associated with older age but also foster the psychological, cognitive, and physical capacities necessary for older adults to thrive amid life’s inevitable stressors.

## 2 Resilience

After the first resilience assessment for older adults was published in 1993, researchers have expanded the concept to encompass multiple dimensions, including psychosocial, physical, and cognitive resilience, leading to a more nuanced understanding of resilience in older populations, but a unified “resilience” definition has yet to be established (Wagnild and Young, 1993). In 2017 NIA Workshop on Measures of Physiologic Resiliencies in Human Aging Resilience defined resilience as the ability to withstand or recover from the negative impact of stressors. It has been stated that resilience is particularly important because it tends to decrease with age, while the risk of encountering stressors increases (Hadley et al., 2017). Low resilience increases susceptibility to stressors, potentially leading to negative outcomes. In contrast, high resilience is associated with more favorable clinical and functional results, making resilience a key target for health maintenance and therapeutic approaches.

**FIGURE 1:**
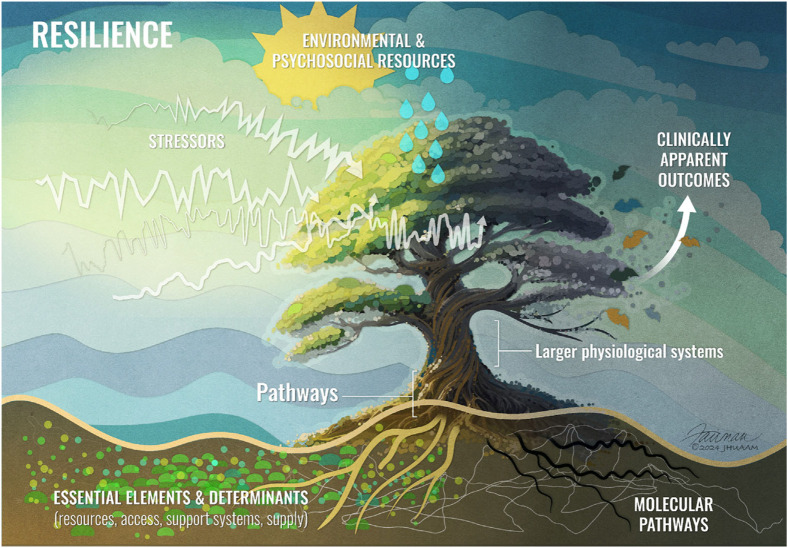
Illustration of Resilience. The biological underpinnings of resilience, including molecular, cellular, and systems biology represented by the tree’s trunk. Social and environmental factors are depicted as the soil. Resilience outcomes across cognitive, physical, and psychological domains are symbolized by the branches of the tree. Figure reused from reference (Colón-Emeric et al., 2025).

In the recent publication of “An Overview of the Resilience World: Proceedings of the American Geriatrics Society and National Institute on Aging State of Resilience Science Conference”, two overarching definitions of resilience was proposed: 1. Attainment of a valued outcome after exposure to a stressor that is expected to diminish that outcome. 2. The capacity, process, or outcome of achieving a valued result after an exposure (Abadir et al., 2023). The conference highlighted that resilience is no longer viewed as a singular construct but rather as a multidimensional phenomenon, encompassing psychosocial, physical, and cognitive components. Each domain reflects different aspects of an individual’s ability to respond to stressors and recover from them, yet these domains are interconnected. Understanding resilience requires an appreciation of how these dimensions interact and how factors such as age, genetics, environment, and life experiences contribute to resilience. In the following workshop in 2024, the ecosystem of resiliency is depicted as a tree, symbolizing how different components contribute to resilience (Figure 1). The tree’s trunk represented as the biological underpinnings of resilience, including molecular, cellular, and systems biology. The soil reflected as the social, environmental, genetic, and psychological factors that nurture resilience biology. These components support the branches of the tree, which represent resilient outcomes across cognitive, physical, and psychological domains, all influenced by acute stressors (Abadir et al., 2023; Colón-Emeric et al., 2025). Building on the original figure, we suggest that reserve may be viewed as a snapshot of the entire tree, representing its current capacity to withstand stressors. It is important to understand how these various domains interact and how resilience is defined in different contexts to ultimately shape a resilient individual.

### 2.1 Reserve and resilience

Physiological reserve is defined as the potential capacity of a cell, tissue, or organ system to function beyond its basal level in response to alterations in physiologic demands by Whitson et al. (2018). In this model, the spectrum from robustness to frailty indicates the level of physiological available to respond to stressors, whereas physical resilience refers to the actualization of that potential (Whitson et al., 2021). Similarly, a 2017 NIA Workshop report described the gap between a system’s baseline function and its maximum response capacity, with the level of reserve setting the threshold at which stress may disrupt system function (Hadley et al., 2017). The amount of reserve influences the threshold of stress a system can endure without disruption. Cognitive reserve, on the other hand, is a characteristic of the brain that allows for cognitive performance that is better than expected given the degree of life-course related brain changes, injury, or disease (Stern et al., 2022). While social reserve was defined as the interpersonal networks and support systems and the ability to be connected to others and society, psychological reserve was defined as a healthy mental state that is free of agitation, anxiety and depression, and other unhealthy mental states previously (Friedland, 2022).

### 2.2 Stressors, adaptability and resilience

In addition to the reserve, the magnitude of stress plays a pivotal role in determining resilience. Resilience is not simply about withstanding stress but about how the body adapts and recovers. The concept of hormesis describes how exposure to low levels of stressors or toxins can trigger adaptive biological responses that enhance health, improve resilience, and even promote longevity. This idea suggests that mild, manageable stressors activate cellular pathways, preparing the body to better handle future, more severe challenges. In aging research, hormesis is particularly relevant, as it highlights how small stressors can improve the body’s ability to resist age-related decline. For example, moderate exercise is a hormetic stressor that induces mild oxidative stress and inflammation (Meng and Su, 2024; Radak et al., 2005). This controlled stress activates protective mechanisms like the AMPK and sirtuin pathways, enhances autophagy, and improves mitochondrial function. These cellular adaptations are thought to contribute to greater overall resilience, improved physiological function, and increased longevity (Militello et al., 2024).

In Whitson et al.’s metaphor, a castle represents physical resilience, with the enemy army symbolizing stressors that challenge the structure (Whitson et al., 2018). The age and condition of the castle, including cracks and missing stones, illustrate frailty. The resilience of the castle depends on its defensive design, structural reserves, and ability to recover quickly from each attack. In the hormesis concept, it could be suggested that smaller, manageable stressors (or minor attacks) could potentially strengthen the castle by revealing weak points, allowing for repairs and preparation for larger assaults. Similarly, in the human body, small stressors may enhance resilience by prompting adaptive responses, ultimately making it stronger and better prepared for future, larger challenges.

In a series of experiments with rats, it was found that exposure to mild restraint or moderate shock stress prior to a more intense shock helped prevent fear responses and shuttle-escape deficits typically seen after severe stress (Plumb et al., 2015; Plumb et al., 2021). Consistent with findings from this animal study, a human study also showed that while chronic stress exposure promotes oxidative damage by frequently and persistently activating the hypothalamic-pituitary-adrenal axis, manageable levels of life stress (eustress) may enhance psychobiological resilience against oxidative damage (Aschbacher et al., 2013). Eustress suggests that low-to-moderate levels of stress could have beneficial effects. This psychological concept has biological parallels in numerous inverted U-shaped relationships, for instance, cortisol demonstrates an inverted U relationship with mitochondrial function, which plays a critical role in managing oxidative stress, studies with cell cultures have shown that short-term, high-dose cortisol boosts mitochondrial performance and offers neuroprotective effects, while prolonged high-dose exposure significantly impairs mitochondrial function and leads to cell death, and low levels of reactive oxygen and lipid species can activate protective pathways, enhancing antioxidant production (Aschbacher et al., 2013; Sapolsky, 1997; Du et al., 2009; Gutierrez et al., 2006).

Similarly, the effects of glucocorticoids on cognitive functions have been found that depend on dosage, exposure duration, and temporal patterns, influencing different stages of memory processing (Li et al., 2019). While chronic exposure to high levels of glucocorticoids is linked to an increased risk of cognitive decline and neurodegeneration, a short-term spike in glucocorticoid levels has been shown to enhance memory consolidation across models (de Quervain et al., 2017; Ouanes and Popp, 2019; Lupien et al., 2005; Meir Drexler and Wolf, 2017). Also, electrophysiological recordings from rat CA1 neurons supported these findings (Diamond et al., 1992). Given the possible age-dependence of stress responses, further research is needed to determine whether similar hormetic patterns are observed across different life stages.

These studies highlight the role of stress intensity and frequency in determining whether stress exposure leads to harm or promotes adaptive, protective responses and the importance of assessing the stressor component in resilience studies. Accurately quantifying both the magnitude of stressors and the perceived stress level is essential for predicting an individual’s resilience and potential outcomes in response to adverse events.

A recent systematic review of conceptual literature identified the core elements of resilience as a stressor, a response, and a mechanism, and emphasized its dynamic nature. Based on differences in the interpretation of how resilience is expressed, the authors distinguished two perspectives: a classical view centered on adaptation to high-intensity stressors and a newer perspective focused on maintaining equilibrium following lower-intensity challenges (Ang et al., 2020). These perspectives may instead represent points along a continuum within a single, dynamic resilience process shaped by the magnitude and duration of stress exposure.

### 2.3 Concept of resilience vs. concept of frailty

Frailty is a key concept in aging research, reflecting increased vulnerability to stress due to diminished physiological reserves. It is commonly conceptualized through two models: physical frailty phenotype and deficit accumulation. The physical frailty phenotype model, widely recognized and developed by Fried et al. (2001), focuses on clinical markers such as weight loss, energy depletion, weakness, slow walking speed, and low physical activity, making it a practical tool for early detection and intervention. Individuals with several of these markers are classified as frail, allowing for targeted interventions like exercise and nutrition to improve physical function. The deficit accumulation model, introduced by Rockwood and colleagues (Rockwood et al., 2005), considers the cumulative effect of various health deficits, including cognitive and psychological impairments.

Frailty and resilience are two key complementary concepts in aging research that, while related, represent distinct perspectives on how older adults respond to stressors (Stenroth et al., 2023). While frailty encompasses a decline in mostly physical systems, resilience, in contrast, has a broader psychosocial focus, incorporating factors such as mental health, coping strategies, social connections, and emotional wellbeing. These psychosocial elements play a crucial role in how individuals adapt to and recover from life’s stressors. Frailty reflects a state of vulnerability which leads to increased susceptibility to adverse outcomes, such as disability and mortality (Fried et al., 2021). Resilience, on the other hand, emphasizes an individual’s ability to adapt and recover from these stressors, focusing on maintaining or regaining health after challenges.

While frailty is often seen as a more static measure of physical decline, resilience is dynamic, highlighting the ability to “bounce back” from stress. Frailty is often described as a syndrome characterized by reduced physiological reserve, leaving older adults more susceptible to adverse outcomes such as falls, cognitive impairment, or functional disability (Fried et al., 2021; Hoogendijk et al., 2019; Evans et al., 2020; De Smet et al., 2020; Theou et al., 2018). Resilience, on the other hand, emphasizes adaptability. It focuses on the ability to adapt and recover from stressors, highlighting the capacity for individuals to maintain or regain function in the face of challenges. It reflects the ability to maintain wellbeing despite adversity (Ang et al., 2020; Zapater-Fajarí et al., 2021; Cesari et al., 2022). Resilient individuals can recover more quickly and effectively from physical, emotional, or social challenges, demonstrating strength in their coping mechanisms.

Despite their differences, frailty and resilience can coexist within the same individual, particularly as people age. The relationship between resilience and frailty across the aging trajectory is dynamic and multifaceted. Frail individuals may still exhibit resilience, particularly through psychological or social strengths, which can mitigate the effects of their physical decline and allow them to cope better with the challenges they face. Conversely, a person who appears physically robust may lack psychological resilience when faced with emotional or social stressors. This interplay underscores the importance of fostering resilience even in those who are frail, as it can lead to better health outcomes and an improved quality of life in aging populations. Understanding both concepts is essential for developing interventions that promote healthy, adaptive aging. A recent study which used self-reported “major health event” (srMHE) to identify resilience showed that resilience and frailty are only partially overlapping concepts. While frailty prevalence among resilient individuals was relatively low, it was still more than double that observed in the control group (those who maintained or improved their performance status without reporting a significant health event). Conversely, around 80% of non-resilient participants were not classified as frail in this study (Pedone et al., 2021). This dynamic interplay across the aging process remains underexplored, and further research is needed to better understand its implications.

Witham and Sayer stated that ‘language matters’ and can create different outcomes (Witham and Sayer, 2015). They mentioned that frailty typically predicts adverse outcomes, signaling that an individual is at high risk for further decline, and it is often used in clinical settings to identify individuals who is at greatest risk of adverse outcomes such as death, dependency, hospitalization or institutionalization (Hoogendijk et al., 2019; Boyd et al., 2005; Topinková, 2008), however, it does not provide room for intervention (Witham and Sayer, 2015). Resilience, however, a measure of how well a person can resist, or recover from, external stressors and predicts positive outcomes. Individuals with higher resilience are more likely to recover from illness or injury, maintain independence, and enjoy a better quality of life as they age. Resilience is not only about avoiding negative outcomes but also about thriving in the face of challenges, leading to better outcomes and potential for interventions (Witham and Sayer, 2015). Later, Rockwood and Mitnitski, emphasized that studying resilience should not replace frailty research, as the two concepts are complementary. They assert that understanding both resilience and frailty will enhance the study of health heterogeneity in aging, and suggest that frailty, remains a critical measure that can predict outcomes and guide interventions (Rockwood and Mitnitski, 2015).

In summary, frailty defined as a condition characterized by reduced physiological reserve, caused by the cumulative aging of multiple organ systems, which leads to higher vulnerability to stressors (Xue et al., 2019). Frailty assessment offers a snapshot of an individual’s physical, functional, and psychosocial reserves, which allows for estimating risks of treatment as well as implementation of supportive interventions that may improve outcomes (Hamaker et al., 2023). Resilience is not directly opposite of frailty (Whitson et al., 2018). It is a more dynamic process which includes subsystems more, and resilience assessment is a recovery assessment rather than a risk assessment. While both concepts are focusing on reserves in relation to aging, each addresses this issue from a different perspective.

### 2.4 Physical resilience

Physical resilience is defined as the ability of the body to recover from or adapt to physical stressors, such as surgery, infection, or injury, rather than psychological or social challenges (Walston et al., 2023). The ability to recover is influenced by available resources (Abadir et al., 2023). It is closely related to but distinct from the concept of frailty, which indicates a diminished capacity to respond to stressors as discussed in detail above. Physical resilience focuses on the body’s ability to activate or recruit resources to maintain stability or return to baseline functioning following a health stressor. Evidence suggests that older adults are generally less capable of enduring physical stress, leading to reduced physical resilience compared to younger individuals. However, the specific biological, physiological, social, and psychological factors underlying this diminished resilience are not fully understood, in part due to significant individual variability in how these factors manifest (Walston et al., 2023).

In clinical settings, measuring physical resilience has important implications for personalized care in older adults. Physical resilience can be predictive of recovery outcomes following acute health episodes, such as hospitalizations or surgeries. Unlike frailty, which is often linked to baseline health deficits, physical resilience is inherently dynamic and represents how effectively an individual can activate and coordinate biological systems to restore function when faced with health challenges. Individuals with strong physical resilience may recover more quickly from an illness or injury compared to those with lower resilience, even if their baseline health is comparable (Abadir et al., 2023). Importantly, objective measures such as hand grip strength have been shown to independently predict clinical outcomes; for instance, lower grip strength is significantly associated with prolonged hospital stays following abdominal surgery, even after adjusting for age, sex, body mass index, frailty, surgical type, and nutritional status (Marano et al., 2022).

Key areas of future research include understanding how physical resilience is supported by various biological systems—such as the cardiovascular, immune, and musculoskeletal systems—and how these systems interact in response to stressors. Additionally, researchers are investigating how resilience changes over the lifespan, and how factors like physical activity, nutrition, and medical interventions can enhance resilience in older adults.

### 2.5 Psychosocial resilience

In the publication of ‘An Overview of the Resilience World: Proceedings of the American Geriatrics Society and National Institute on Aging State of Resilience Science Conference’, Masten’s definition of resilience “the capacity of a dynamic system to adapt successfully to disturbances that threaten system function, viability, or development” was restated from the perspective of psychological science, particularly developmental and life span psychology (Abadir et al., 2023; Masten, 2014). It is reported that this broad definition is applicable across various research disciplines. Psychosocial resilience pertains to the capacity to adapt to social and emotional stressors throughout the lifespan. It is not only a response to acute challenges like bereavement or illness but also to chronic adversities such as social isolation or long-term health conditions. Psychosocial resilience can be seen as a dynamic process, shaped by an individual’s interactions with their environment, relationships, and internal coping mechanisms.

Psychosocial resilience is not a uniform trait but can vary across different life domains. Individuals may show high resilience in managing social relationships but may struggle with emotional regulation or *vice versa*. This variation underscores the importance of a nuanced understanding of resilience, especially in older adults who may face complex social and psychological challenges. Longitudinal studies are needed to more accurately capture the dynamic nature of psychosocial resilience over time. This method would allow for a deeper understanding of how short-term stress responses transition into long-term adaptive mechanisms.

A key aspect of psychosocial resilience is its higher potential for growth, enabling individuals to become more resilient than they were before encountering a stressor. This characteristic sets it apart from the other two resilience domains (Abadir et al., 2023) and might suggest that the minor stress faced over a lifetime builds resilience. A study revealed that older adults had high overall resilience, which significantly benefited their mental health (Upasen et al., 2024). This may indicate that their accumulated experiences of overcoming challenges throughout life contribute to their heightened resilience, helping them manage and mitigate future adversities more effectively. Experiencing stress early in life can enhance resilience in later years, whereas a lack of stress exposure may increase vulnerability to challenges (Li et al., 2019).

Behavioral and social factors are integral to the aging process. Prolonged exposure to toxic stressors can accelerate aging by directly influencing biological aging processes or contributing to harmful behaviors, which exacerbates socioeconomic disparities in aging. In contrast, hormetic stressors—brief, moderate-intensity stressors—can promote stress resilience, enabling quicker recovery and even potential rejuvenation of cells and tissues. This distinction highlights how different stress types can either worsen or improve the resilience depending on their nature and intensity (Epel, 2020).

Some of the qualities that enable older adults to adapt to the challenges of aging have been listed as positive interpersonal relationships, a strong sense of self-efficacy, and positive self-esteem, which contribute to their ability to navigate difficulties, a sense of purpose, spirituality, and the ability to use humor and creativity in challenging situations (Resnick et al., 2020; Bonanno et al., 2007; Bolton et al., 2016). Acceptance of physical and mental changes, maintaining a positive attitude, and effectively identifying and using resources are also suggested as key factors that support older adults in overcoming adversity, along with traits such as self-determination, optimism, grit, and seeing joy in each day. Resilient individuals described as who have adaptive coping styles, derive meaning from their experiences, and rely on prior hardships to guide their responses to new challenges (Resnick et al., 2020). Other notable suggested characteristics for resilient older adults include self-acceptance, the ability to keep things in perspective, and taking care of their physical and emotional wellbeing (Resnick et al., 2020). A recent study found that higher social support, optimism, and sense of control were significantly associated with lower response to chronic stress (An and Kwon, 2023). A recent meta-analysis in community-living older adults identified several key personal and contextual factors significantly associated with resilience in older adults. Resilience was linked with higher levels of optimism, purpose in life, self-efficacy, self-transcendence, sense of coherence, and a health-promoting lifestyle. Moderate associations were also observed with life satisfaction, psychological wellbeing, morale, and physical and mental self-rated health. On the other hand, depression, loneliness, psychological distress, and experience of stigma were moderately associated with lower resilience. Among contextual factors, social support, income, education, and the size of social networks were positively related to resilience, though these associations were generally weaker (Górska et al., 2021).

### 2.6 Cognitive resilience

As individuals age, cognitive decline typically occurs across various abilities, though the rate and extent of this decline vary significantly between individuals. Some people experience sharp cognitive deterioration, while others manage to maintain their cognitive performance well into later life. Although many factors influence these different aging trajectories, certain individuals seem to be more resilient to the adverse effects of aging and related pathological changes than others (Stern et al., 2019). The concept of reserve capacity was first observed in patients who exhibited extensive neural damage without the expected corresponding functional impairments (Katzman et al., 1988). This led to the idea that individuals with larger brain volumes, greater brain mass, or a higher number of neurons might have a protective advantage against cognitive decline, a phenomenon attributed to a higher “brain reserve” capacity. This reserve allows the brain to compensate for damage, maintaining cognitive function despite significant neural loss. Then, in 2002, “cognitive reserve” concept first conceptualized, suggesting that the brain actively attempts to cope with brain damage (Stern, 2002).

Cognitive resilience defined as a broad, umbrella concept used to address the challenges associated with aging and disease (Stern et al., 2020). This concept integrates several related ideas, including brain maintenance and cognitive reserve, all of which contribute to an individual’s ability to cope with neurological and physiological changes over time. Cognitive resilience refers to the brain’s ability to maintain or recover cognitive function despite aging-related changes or neuropathological damage. Cognitive resilience is closely related to two concepts: cognitive reserve and brain maintenance. Cognitive reserve is the brain’s capacity to utilize alternative neural pathways or mechanisms to cope with damage, while brain maintenance involves the preservation of neural integrity over time. “Cognitive reserve” refers to the brain’s ability to maintain higher-than-expected cognitive performance despite aging-related changes, brain injuries, or diseases. For instance, individuals with high cognitive reserve may show minimal cognitive symptoms despite the neuronal damage caused by conditions like Alzheimer’s disease. Factors contributing to cognitive reserve often include higher levels of education and occupational achievement.

“Brain maintenance,” on the other hand, involves the preservation of neural resources or the absence of neuropathological changes over time, which helps sustain cognitive function in older adults. The term “maintenance” refers to the concept that certain lifestyle factors, such as regular physical activity, can help preserve brain health. This is achieved by slowing down age-related brain changes and enhancing the brain’s natural repair processes, thereby promoting overall neural integrity (Alvares et al., 2022).

In cognitive resilience research, the focus is on recovery from stressors, particularly on maintaining cognitive function and performance. Both cognitive reserve and brain maintenance are shaped by genetic and environmental influences that act throughout an individual’s life. In the literature on cognitive health, “resilience” encompasses both cognitive reserve and brain maintenance. Factors that contribute to cognitive resilience include educational attainment, intellectual engagement, social interaction, and physical activity. For example, individuals with higher levels of education or more cognitively stimulating occupations tend to have greater cognitive reserve, allowing them to cope better with cognitive decline or neurodegenerative conditions like Alzheimer’s disease (Abadir et al., 2023).

Current research focuses on identifying the genetic and lifestyle factors that enhance cognitive resilience. Studies also emphasize the need for longitudinal research to track how cognitive resilience evolves over time and in response to environmental exposures or life events. Additionally, animal studies and advanced neuroimaging techniques are being used to explore the molecular and neural mechanisms underlying cognitive resilience, offering potential pathways for therapeutic interventions. Cognitive resilience is not the result of a single stressor but rather a response to cumulative damage over many years. In this context, resilience is characterized by having a high cognitive reserve, while low reserve indicates reduced resilience to neuropathology (Abadir et al., 2023).

The term “resistance” refers to the ability to avoid pathology altogether, such as remaining free from significant Alzheimer’s Disease (AD) pathology. In contrast, “resilience” is generally used to describe coping with pathology, meaning an individual can maintain normal cognitive function despite the presence of AD-related brain changes. In summary, resilience is closely tied to the concept of cognitive reserve, or “coping with pathology,” while resistance is associated with the absence or delay of brain changes, linked to the idea of brain maintenance (Table 1) (Alvares et al., 2022).

**TABLE 1:** Key terms and definitions.

Key terms and definitions	
Resilience	The ability to withstand or recover from the negative impact of stressors (Hadley et al., 2017).
Frailty	Increased vulnerability to stress due to diminished physiological reserves (Fried et al., 2001).
Cognitive Reserve	The characteristic of the brain that allows for cognitive performance that is better than expected given the degree of life-course related brain changes, injury, or disease (Stern et al., 2022).
Physiological reserve	The potential capacity of a cell, tissue, or organ system to function beyond its basal level in response to alterations in physiologic demands (Whitson et al., 2018).
Psychological reserve	The ability to maintain healthy mental function and avoid agitation, anxiety, depression, and other unhealthy mental states (Friedland, 2022).
Social reserve	The interpersonal networks and support systems and the ability to be connected to others and society (Friedland, 2022).
Psychological Resilience	The capacity of a dynamic system to adapt successfully to disturbances that threaten system function, viability, or development (Abadir et al., 2023; Masten, 2014).
Cognitive resilience	Refers to the brain’s ability to maintain or recover cognitive function despite aging-related changes or neuropathological damage (Stern et al., 2020).
Physical resilience	The ability of the body to recover from or adapt to physical stressors, such as surgery, infection, or injury, rather than psychological or social challenges (Walston et al., 2023).
Brain maintenance	The preservation of neural resources or the absence of neuropathological changes over time, which helps sustain cognitive function in older adults (Alvares et al., 2022).

While resilience is typically associated with a lower risk of developing dementia, it paradoxically may also be linked to a faster rate of cognitive decline and increased mortality once Alzheimer’s disease is diagnosed. This rapid decline could occur because the brains of highly educated individuals, who generally show greater cognitive resilience, endure more significant functional and structural damage before the symptoms of the disease become evident. This gap between the extent of brain damage and cognitive performance suggests that these individuals have robust compensatory mechanisms that manage to mask the disease for longer. However, once these mechanisms are overwhelmed, cognitive decline accelerates rapidly, pushing the system past its breaking point (Stern et al., 2019).

### 2.7 Change in resilience across lifespan

Understanding resilience requires focusing on how individuals dynamically respond to stress rather than viewing resilience as a fixed trait. It involves assessing the flexibility and adaptability of physiological and psychological systems when exposed to stressors. Instead of examining every data point in a trajectory, it has been suggested to use models to capture trajectories and show the extent of improvement over time after dysregulation. This approach allows for a deeper understanding of how resilience manifests and adapts over time (Abadir et al., 2023). Moreover, resilience is considered a dynamic trait that can fluctuate over time, rather than consistently resulting in positive outcomes. Instead of labeling individuals as resilient or not, it is more effective to focus on identifying the factors that strengthen resilience and improve one’s capacity to handle stress. The concept of post-traumatic growth is also gaining traction, with growing evidence indicating that challenging experiences can foster personal development. In the context of resilience, maintaining baseline functioning is viewed as success, while failure is defined by an inability to return to that baseline. Regular assessments may show that individuals who initially deteriorate could ultimately experience better long-term outcomes compared to those who remain unchanged (Abadir et al., 2023).

A study examining psychological resilience across age groups found that older adults demonstrated greater resilience, particularly in emotional regulation and problem-solving abilities. In contrast, younger individuals showed higher resilience in areas related to social support. Regardless of age, low resilience was associated with poor self-rated health and low energy levels (Gooding et al., 2012). Interestingly, a study investigating age-related differences in HPA axis resilience found no significant differences in hormonal responses to stress following exposure to the Trier Social Stress Test (TSST) in healthy older adults (Kudielka et al., 2000).

## 3 How should we measure resilience?

### 3.1 Resilience biology

Biological resilience in older adults depends on the integrity of bodily systems, which is sustained by the body’s ability to preserve its complex communication and regulatory networks that support homeostatic balance. These pathways are crucial for managing stress and maintaining stability across various physiological functions. The accumulation of age-related defects at the molecular and cellular levels contributes to diminished resilience (Colon-Emeric et al., 2023). These defects include issues like stem cell exhaustion, mitochondrial dysfunction, immune system dysregulation, and changes in nutrient sensing. Additionally, aging-related environmental, social, and psychological factors can significantly influence how older adults recover from health stressors. There is also considerable variability in how quickly and to what extent older adults recover from similar stressors, highlighting the heterogeneity in resilience among this population (Abadir et al., 2023; Hamaker et al., 2023; Colon-Emeric et al., 2023).

The biological and potential molecular foundations of frailty include various interconnected factors such as metabolic dysfunction, chronic inflammation, impaired function of the hypothalamic-pituitary-adrenal axis, dysregulation of energy homeostasis, endocrine imbalances, mitochondrial dysfunction, oxidative stress, epigenetic changes, and genomic instability. Many of these factors are interrelated and contribute to the gradual loss of physiological capacities over time, ultimately depleting the body’s reserves. This raises the question of whether these processes are analogous to or distinct from the mechanisms that govern resilience. In align with this, a study found that biomarkers of inflammation, metabolic and mitochondrial function, and epigenetic dysregulation explain 27% of the variance in the expected recovery differential that captures the difference between actual recovery and predicted recovery after hip fracture (Dc et al., 2020). Understanding the overlap and distinctions between frailty and resilience could provide insights into how individuals either maintain or lose their ability to recover from stressors as they age (Abadir et al., 2023). It was shown that frailty index based on health deficits are more effective than DNA methylation-based age metrics in predicting biological age (Kim et al., 2017). While frailty indices can serve as indicators of biological aging, frailty itself may emerge too late in the aging process to be a useful marker of resilience (Kim et al., 2017).

Data from the Religious Orders Study and the Rush Memory and Aging Project have also been used to explore the molecular mechanisms of resilience in brain health. These data show that some people experience rapid cognitive decline, a few have a slower decline, and some have no cognitive decline. In one analysis, 10 of 11 pathological indices examined (including markers of Alzheimer’s disease, other neurodegenerative diseases, and cerebrovascular conditions) were associated with faster decline and accounted for 2%–34% of the variation in decline. But more than 50% of the variations in cognitive decline were not explained by the pathologic indices examined (Boyle et al., 2021; Boyle et al., 2019). Although age-related neuropathologies explain a significant portion of the variation in cognitive decline in later life, a substantial amount of variability remains unaccounted for, even after considering a broad range of neuropathological factors. These results underscore the complexity of cognitive aging and emphasize the need for continued research to define other factors contributing to resilience.

Over the past four decades, “omics” has evolved into a key research tool and methodology for systematically studying disciplines like life sciences and medicine. By examining the shifts in health reserves and risk defense mechanisms over time, and using entire populations of regions or countries as a unit of study, omics provides a broad perspective on human aging at a macro level (Zheng and Guo, 2022). A National Heart, Lung, and Blood Institute workshop report on resilience in cardiovascular health and wellness highlights the need to expand biomedical research to uncover the genetic, molecular, and signaling mechanisms that support resilience (Taylor et al., 2022). It has been suggested that key to advancing this research would be the integration of computer modeling and bioinformatics, which will help decode complex datasets, generate hypotheses, and predict outcomes. The workshop highlighted that advancements in omics technologies will be instrumental in accelerating the identification of gene networks and molecular interactions that contribute to resilience (Taylor et al., 2022). A shift from examining single genes to studying the interplay of gene networks was suggested, as these networks create feedback systems that enhance adaptability. Additionally, emerging tools like organ-on-a-chip technologies and new disease models will improve our understanding of how resilience operates on a biological level. To better evaluate resilience, they suggest establishing standardized biological and physiological markers. These would provide objective measurements, particularly in assessing genetic and non-genetic mechanisms that allow organisms to adapt to environmental, chemical, or pathogen-related stressors. According to this report, key molecular mechanisms driving resilience, such as reactive oxygen species (ROS), epigenetic modifications, and ligand-activated transcription factors, may play crucial roles in the adaptive processes that define resilience. The report envisioned that cloud-based data sharing platforms could further accelerate research efforts by facilitating global collaboration and data access (Taylor et al., 2022).

### 3.2 Conceptualization of resilience measurement

As the global population of older adults rapidly increases (Ismail et al., 2021), measuring resilience is vital, as it can enhance shared decision-making and refine targeted interventions. Moreover, resilience predictors may offer deeper insights into the biological foundations that shape these outcomes. It is critical to develop better tools for assessing this resilience (Schorr et al., 2018).

Both traditional and emerging conceptual frameworks are being employed to enhance the understanding of physical resilience and its biological underpinnings (Walston et al., 2023; Colon-Emeric et al., 2023). Conventional epidemiological methods in clinical and population studies are instrumental in identifying risk factors that contribute to non-resilient phenotypes and adverse clinical outcomes following various physical stressors (Barbara et al., 2011; Franceschi et al., 2018; Varadhan et al., 2018). Chronological age, disease burden, cognitive and functional status prior to a stressor, along with biomarkers, were identified as potential predictors of resilience (Walston et al., 2023). While epidemiological studies have identified factors linked to physical resilience, they haven't fully explained the biological mechanisms that drive a resilient response. It is believed that resilience depends on interactions among physiological systems that maintain homeostasis—a state of balance managing external stressors. Since these systems are responsive and adaptive, studying resilience in a resting state might not provide valuable insights. Instead, experiments where controlled stimuli are introduced, and the body’s physiological responses are measured, are crucial for understanding the capacity for resilience (Walston et al., 2023). And, because of physical resilience’s dynamic nature, repeated outcome measurement over time following a particular health stressor are also suggested in resilience studies (Cesari et al., 2022; Colon-Emeric et al., 2023).

The Trans National Institutes of Health (NIH) Resilience Working Group defined “resilience” as a system’s capacity to resist, recover better (grow), or adapt in response to a challenge or stressor (National Institutes of Health, 2024). This system can refer to various domains, such as individual or community levels, and spans different processes including social, behavioral, and physiological responses. Over time, a system’s resilience may fluctuate depending on factors such as the severity and duration of exposure to a stressor, as well as the system’s intrinsic characteristics. The NIH Resilience Research Design Tool was developed to standardize the design and reporting of resilience studies across different contexts (National Institutes of Health, 2024). Also, two conceptual models mentioned at the 2022 NIA Conference—one from the Johns Hopkins Pepper Center and another from Duke Pepper Center—offer frameworks for studying physical resilience, particularly in relation to functional recovery after health stressors (Abadir et al., 2023). The Hopkins model focuses on the capacity of physiological systems to manage pre- and post-stressor functional responses. In this model, a resilient system may experience some functional decline but still retains its essential functionality. The Study of Physical Resilience and agING (SPRING) is investigating individuals undergoing one of three major medical stressors: total knee replacement, bone marrow transplantation for hematologic cancers, or the start of dialysis. Participants are evaluated about a month prior to the procedure to establish their baseline physical resilience. After the stressor, researchers measure the participants’ resilience by tracking various indicators such as frailty, physical function, cognitive performance, and outcomes like hospitalization or mortality (Walston et al., 2023). The Duke model presents resilience as a dynamic process that allows a system to regain equilibrium following a stressor (Whitson et al., 2021). This model emphasizes the importance of pre-stress reserves, which encompass psychological, physiological, and cognitive domains, in determining how effectively a person adapts to health challenges. In the NIH conference paper, the example of COVID-19 vaccinations was used to illustrate how interventions can enhance physiological reserves and bolster resilience (Abadir et al., 2023).

Cognitive reserve, as a theoretical construct, cannot be directly measured. However, three primary approaches are commonly used to measure cognitive reserve: socio-behavioral indicators, residual approaches, and functional neuroimaging studies (Stern et al., 2020). In this framework, research on cognitive reserve should encompass three core elements: the brain’s condition (indicating structural changes or pathology), clinical or cognitive performance (highlighting the effects of brain damage), and socio-behavioral indicators of cognitive reserve (such as indices of lifelong experiences). In summary, refining theoretical constructs while simultaneously developing reliable and valid indicators is essential to advancing our understanding of resilience and leveraging it to improve cognitive trajectories in older adults. Since resilience develops over a lifetime, it is crucial to explore its developmental origins and identify risk factors that influence its evolution. Longitudinal studies spanning the life course are needed to clarify how brain reserve forms, how cognitive reserve is constructed, how brain maintenance operates, and how compensatory mechanisms are activated. Furthermore, age-related changes in plasticity and the capacity to handle systemic and environmental challenges are especially relevant in understanding resilience in aging (Stern et al., 2019).

There are several scales to measure psychosocial measures of resilience such as the Brief Resilience Scale (BRS) which assesses resilient outcomes (Smith et al., 2008), Connor-Davidson Resilience Scale (CD-RISC) which understands resilience as a personality trait and assess a compound of resilience factors (Velickovic et al., 2020), Hardy-Gill Resilience Scale which assesses resilience as a coping process in response to a specific life event (Hardy et al., 2004), Resilience Appraisals Scale which assess appraisals of their ability to cope with emotions, solve problems, and gain social support (Gooding et al., 2012; Johnson et al., 2010), Ego Resilience Scale (Block and Kremen, 1996), Essential Resilience Scale (Chen et al., 2016), Physical Resilience Scale (PRS) (Barbara et al., 2011), Resilience Scale which evaluates resilience as a personality trait (Resnick and Inguito, 2011), Resilience Scale for Adults (RSA) (Friborg et al., 2006), and Scale of Protective Factors (SPF) (Ponce-Garcia et al., 2015). Among many psychosocial resilience assessment scales, these four are reported as mostly used ones: Connor-Davidson Resilience Scale (CD-RISC), Connor-Davidson Resilience Scale 10 (CD-RISC 10), Resilience Scale for Adults (RSA) which is also an assessment tool of protective factors, and Brief Resilience Scale (BRS) (Salisu and Hashim, 2017). In another systematic review, The Connor-Davidson Resilience Scale (CD-RISC) and the Resilience Scale were found as the most used scales (Ferreira et al., 2021).

### 3.3 Stress test

To effectively assess resilience, it is essential to evaluate an individual’s capacity to respond to stress before an actual adverse event occurs such as how a cardiac stress test evaluates cardiovascular function. Identifying resilience before a major health event such as a hip fracture could lead personalized interventions and improve outcomes in older adults. However, unlike cardiac stress tests, we currently lack standardized, domain-specific tools to evaluate resilience and the risk of functional decline prior to real-life stressors.

Existing biomarker-based tests, such as ACTH stimulation for endocrine function or glucose tolerance testing for metabolic resilience, provide some insight into physiological reserves, but they are not comprehensive (Colón-Emeric et al., 2025). Furthermore, emotional and psychological stressors such as grief and social isolation also significantly impact resilience, especially in older populations. While some tests such as Trier Social Stress Test (TSST) and habituation of acoustic startle were used (Zapater-Fajarí et al., 2021; Kudielka et al., 2000; Walker et al., 2017; Nalivaiko et al., 2017), standardized protocols to simulate and assess resilience to emotional stress are currently lacking, despite their relevance to health outcomes.

To advance the field, future research should focus on developing validated, multidimensional stress testing protocols that encompass not only physical but also emotional and cognitive domains. Such tools are critical for identifying at-risk individuals and designing targeted, preventative strategies to maintain or enhance resilience across the lifespan.

## 4 Multimodal interventions

Physical, psychosocial, and cognitive resilience are deeply interconnected and influenced by the underlying reserves within each domain. Physical stressors such as illness, injury, or frailty can compromise psychological coping abilities and cognitive functioning. Similarly, psychosocial adversity, including chronic stress or social isolation, may impair physiological recovery and diminish cognitive reserve. In turn, declines in cognitive resilience can reduce an individual’s capacity to respond effectively to physical challenges or sustain social engagement. These interdependencies highlight the importance of conceptualizing resilience as a multidimensional process that operates across systems and evolves throughout the life course. Accordingly, interventions targeting one domain such as physical exercise, social engagement, or cognitive training may have beneficial cross-domain effects, reinforcing resilience across multiple systems (Du et al., 2025; Abo and Hamaguchi, 2024).

Enhancing resilience can significantly reduce negative reactions to stress, lower the likelihood of developing health issues, and improve the mood and overall quality of life in older adults. As such, developing strategies to boost resilience is crucial and urgently needed to promote better health outcomes for this population (Kiosses and Sachs-Ericsson, 2020). Some individuals may have a genetic predisposition for resilience, while others cultivate it through life experiences or targeted interventions (Resnick et al., 2020). Boosting resilience in older adults can be approached through several strategies, both environmental and intrinsic. To enhance overall resilience, it is necessary to target mechanisms that affect multiple systems (Witham and Sayer, 2015).

As mentioned above, the stress level is really critical. While low to moderate stress can build resilience, excessive or chronic stress can overwhelm the body’s adaptive capacity, leading to negative health outcomes (Li et al., 2019). This balance is essential in aging, where the body’s resilience naturally decreases over time. Regular physical activity, including aerobic and resistance exercises, has been shown to not only slow down age-related decline but also to strengthen muscles, bones, and other systems, allowing the body to adapt to stress more effectively (Militello et al., 2024; Resnick et al., 2020; Ji et al., 2016). As such, physical activity is a key strategy in building and maintaining resilience throughout life. However, there are still no standardized guidelines specifying the optimal duration and frequency of physical activity and nutritional intake needed to effectively enhance physical resilience in older adults.

Although the optimal exercise type and dosage for promoting resilience in older adults remains under investigation, extensive research supports the beneficial effects of physical activity on aging and mortality, with health gains strongly tied to both the intensity and volume of exercise (Izquierdo and Fiatarone Singh, 2023). Crucially, the modality of exercise should be selected based on specific needs. In a precision-exercise medicine model, factors like duration, intensity, and modality should be personalized to improve adherence and optimize outcomes. Multicomponent programs that incorporate resistance, aerobic, balance, and mobility exercises have shown superior results, particularly when mimicking real-life tasks such as sit-to-stand movements (Cadore et al., 2019). For frail or cognitively impaired individuals, combining physical training with dual-task cognitive exercises can enhance both motor and cognitive domains of resilience (Izquierdo and Fiatarone Singh, 2023). Additionally, a randomized controlled trial involving a primary care intervention for older adults found that a 3-month home-based strength-focused exercise program, paired with dietary guidance to achieve 1.2 g/kg/day of protein intake, significantly reduced frailty and improved self-reported health (Travers et al., 2023).

For physical resilience, some efforts in addition to diet and exercise have been suggested to buffer against precarious conditions, such as removing inappropriate medications that could trigger harmful cascades of health issues (Witham and Sayer, 2015). Psychological stress and distress have been associated with increased levels of oxidative damage. Internally, reducing excessive oxidative stress responses following injury is essential for enhancing resilience (Aschbacher et al., 2013). A significant feature of aging-related diseases is the “knock-on” effect, where stress on one system can negatively impact multiple other systems. This interaction explains why a majority of secondary hospital admissions occur due to a different illness than the initial one (Witham and Sayer, 2015). Therefore, focusing on resilience in a single organ system is insufficient for the whole body. Additionally, interventions that have wide-ranging effects, such as those targeting bone, muscle, cardiovascular, and cognitive function simultaneously, are more likely to be effective in promoting resilience in older adults (Witham and Sayer, 2015). Vaccination has given as an example above, which enhances external resistance to threats by building immunity (Abadir et al., 2023).

Some strategies also have been suggested to build psychosocial resilience such as enhancing self-efficacy and self-esteem, participating in social activities, maintaining a positive and optimistic outlook, using humor, and embracing change as an opportunity for growth (Table 2) (Resnick et al., 2020). Additionally, seeking support from others, giving back to the community, and incorporating spiritual or creative practices can further strengthen resilience. Actively working to improve resilience can lead to significant benefits in both physical and psychological health, potentially reducing the impact of disease and lowering morbidity and mortality. This process requires an understanding of resilience as a dynamic, multi-level phenomenon shaped by individual, family, and community factors, as well as life circumstances and available resources (Resnick et al., 2020). Additionally, fostering strong social networks and providing timely healthcare and social services are crucial for reducing vulnerability (Witham and Sayer, 2015). A meta-analysis found that all three categories of interventions (1) cognitive behavioral therapy (CBT)-based interventions, (2) mindfulness-based interventions, and (3) mixed interventions combining CBT and mindfulness had a positive effect on resilience, with an overall effect size of 0.44 (95% CI: 0.23 to 0.64). Subgroup analyses indicated that CBT-based, mindfulness, and mixed interventions were all effective (Joyce et al., 2018). Lastly, digital interventions based on different approaches, such as CBT and mindfulness, have been shown to be effective in promoting psychosocial resilience (Schäfer et al., 2024).

**TABLE 2:** A summary of assessment methods and possible interventions for each resilience domain.

	Physical	Psychosocial	Cognitive
Assessment Tests for Measuring Resilience (Provocative) and Reserve	Short physical performance battery (Walston et al., 2023) Grip Strength (Whitson et al., 2021) Three-Minute Walk Test (Whitson et al., 2021) Timed Up and Go Test (Kian and Chang, 2025) Gait speed dual task test (O’Brien and Holtzer, 2021) *In-vitro* PBMC response to LPS/vaccine (Whitson et al., 2021) Self-reported “major health event” (Pedone et al., 2021) Standing balance and displacement of the center of pressure (COP) (Manning et al., 2025) Physical Resilience Scale (PRS) (Barbara et al., 2011) Heart rate variability (Whitson et al., 2021) 36-Item Short Form survey (Laskow et al., 2022)	Brief Resilience Scale Connor-Davidson Resilience Scale (Smith et al., 2008; Velickovic et al., 2020) Ego Resilience Scale (Block and Kremen, 1996) Essential Resilience Scale (Chen et al., 2016) Physical Resilience Scale (Barbara et al., 2011) Resilience Scale for Adults (Friborg et al., 2006) Scale of Protective Factors (Ponce-Garcia et al., 2015) Wagnild and Young Resilience Scale (Wagnild and Young, 1993) Hardy-Gill Resilience Scale (Hardy et al., 2004) Resilience Appraisal Scale (Gooding et al., 2012) Trier Social Stress Test (TSST) (Kudielka et al., 2000) Habituation of acoustic startle (Nalivaiko et al., 2017)	The Cognitive Reserve Unit Scale (Joshi and Galvin, 2022) Battery for the Assessment of Cognitive Reserve (Nogueira et al., 2023) Near-Infrared Spectroscopy - Cerebrovascular Reactivity (Whitson et al., 2021) 3MS (Whitson et al., 2021) Trails making test A/B (Whitson et al., 2021) Fifteen item word list (Whitson et al., 2021) Digit symbol substitution test (Whitson et al., 2021)
Possible Interventions for Improving Resilience and Reserve	Regular exercise and nutrition (Godos et al., 2025; Azzolino et al., 2021) (Militello et al., 2024; Ji et al., 2016) Strength training (Manini and Pahor, 2009) Immunization (Abadir et al., 2023) Mind-body approaches (Wu et al., 2023)	Counseling, support groups, mindfulness, stress management techniques, building strong social networks (Resnick et al., 2020) (Witham and Sayer, 2015) Digital Interventions (Schäfer et al., 2024)	Cognitive training (Ball et al., 2002) Education (Stern et al., 2020) Lifestyle modifications (diet and exercise) (Song et al., 2022) Positive behaviors (mindfulness, optimism, self-efficacy) (Joshi and Galvin, 2022) Dual task intervention (Abo and Hamaguchi, 2024; Ali et al., 2022; Versi et al., 2022)

For cognitive resilience, attention should be directed to various factors that influence brain aging and modulate resilience, including physical fitness, social engagement, and risk factors related to vascular health, metabolism, and neuroinflammation. Additionally, genetic variations associated with risk, socioeconomic deprivation, chronic stress, and environmental pollution are critical considerations in shaping the resilience of the aging brain (Stern et al., 2019; Stern et al., 2020). Recently, the beneficial effects of dual-task interventions, which include both physical exercises and cognitive tasks performed simultaneously, have been demonstrated in improving motor function and cognition (Abo and Hamaguchi, 2024; Ali et al., 2022; Versi et al., 2022).

The concept of the exposome, which encompasses all environmental exposures that a person encounters throughout life, has recently gained attention as a critical factor in resilience. Environmental exposures, such as air pollution, socioeconomic status, neighborhood conditions, and social inequities, play a significant role in determining an individual’s resilience across all domains. Research indicates that individuals living in disadvantaged environments may exhibit lower resilience due to chronic stressors, environmental toxins, and limited access to resources that promote health and recovery (Abadir et al., 2023). The exposome can also include positive factors that enhance resilience, such as social support networks, community engagement, and access to healthcare (National Research Council (US), Institute of Medicine (US), 2013). Understanding how different aspects of the exposome interact with biological and psychological factors throughout the life course is essential for developing interventions that promote resilience in vulnerable populations.

Tailoring interventions based on genomic and physiological besides contextual factors is essential to maximize effectiveness. For instance, individuals carrying the APOE ε4 allele or those with high inflammatory or oxidative stress markers may respond better to early lifestyle modifications that include anti-inflammatory diets, aerobic activity, or stress-reduction techniques (O’Shea et al., 2024). Intervention strategies should also be adapted to an individual’s functional status and health conditions. Robust older adults may participate safely in higher-intensity, group-based exercise programs, whereas prefrail or frail individuals may benefit more from home-based, low-impact routines such as supervised telerehabilitation. In parallel, dietary interventions may require modification for those with comorbidities such as diabetes, renal disease, or malnutrition. Taken together, advancing resilience-based care requires not only identifying core strategies, but also customizing their delivery to align with each individual’s genetic risk, physiological reserve, health profile, and cultural setting.

A systematic review evaluating 43 randomized controlled trials aimed at fostering resilience identified major methodological and conceptual limitations. These included inconsistent or missing definitions of resilience, varied outcome measures, limited assessment of individual stressor exposure, lack of sample size calculations, inadequate control groups, and insufficient baseline diagnostics as well as a general absence of long-term follow-up and adverse event monitoring. These findings emphasize the importance of reaching a consensus on how resilience is defined and operationalized in intervention studies to advance methodological rigor and stimulate further progress in this growing field (Chmitorz et al., 2018).

## 5 Current knowledge gaps and future directions

Resilience in aging is a complex and multifaceted phenomenon, involving psychosocial, physical, and cognitive dimensions. As the older adult population continues to grow, it becomes increasingly important to understand the mechanisms of resilience across these domains. Such knowledge is essential for developing effective interventions aimed at improving health outcomes and overall quality of life. While resilience research has made considerable progress, significant knowledge gaps remain. These gaps hinder the ability to fully harness resilience as a means to promote healthy aging and to tailor interventions that address the unique challenges faced by older adults. Future research should focus on several critical areas to advance the field (Abadir et al., 2023).

First, dynamic longitudinal studies are needed to capture the evolving nature of resilience in older adults. Tracking individuals over time and across various stressors will provide valuable insights into how resilience operates. These studies should integrate data from multiple domains—physical, psychological, and cognitive—allowing for a more comprehensive understanding of the resilience process. Personalized interventions that enhance resilience should also be prioritized. Tailoring strategies such as physical activities, cognitive training programs, and strengthening social support networks to individual needs will be key to supporting older adults in maintaining resilience. Such approaches recognize the diversity of aging experiences and allow for targeted care that addresses specific vulnerabilities. A multidisciplinary approach will be crucial for advancing resilience research. Collaboration between fields such as biology, psychology, sociology, and computational science will provide a more holistic understanding of how resilience can be nurtured across the lifespan. By combining different perspectives, researchers can develop interventions that consider the broader biological, social, and psychological factors influencing resilience.

Finally, integrating the concept of the exposome—lifelong environmental exposures—into resilience research will be essential. Understanding how these exposures shape resilience will help identify at-risk populations and enable the development of interventions that mitigate the long-term effects of chronic stressors. This will allow for more proactive, tailored strategies to support resilience in older adults.

## 6 Conclusion

In conclusion, resilience plays a pivotal role in determining how older adults respond to and recover from stressors. While considerable progress has been made in understanding resilience across various domains, much remains to be learned about the dynamic processes that support it. By addressing these gaps through comprehensive research and personalized interventions, we can contribute to improving the health and wellbeing of the aging population.
